# Forced oscillations and respiratory system modeling in adults with cystic fibrosis

**DOI:** 10.1186/s12938-015-0007-7

**Published:** 2015-02-13

**Authors:** Adma N Lima, Alvaro C D Faria, Agnaldo J Lopes, José M Jansen, Pedro L Melo

**Affiliations:** Pulmonary Function Laboratory - Faculty of Medical Sciences, State University of Rio de Janeiro, Rio de Janeiro, Brazil; Biomedical Instrumentation Laboratory - Institute of Biology and Faculty of Engineering, State University of Rio de Janeiro, Rio de Janeiro, Brazil; BioVasc Research Laboratory - Institute of Biology, State University of Rio de Janeiro, Rio de Janeiro, Brazil

**Keywords:** Respiratory system modeling, Biomedical instrumentation, Diagnosis, Cystic fibrosis, Adults, Respiratory biomechanics, Forced oscillation technique

## Abstract

**Background:**

The Forced Oscillation Technique (FOT) has the potential to increase our knowledge about the biomechanical changes that occur in Cystic Fibrosis (CF). Thus, the aims of this study were to investigate changes in the resistive and reactive properties of the respiratory systems of adults with CF.

**Methods:**

The study was conducted in a group of 27 adults with CF over 18 years old and a control group of 23 healthy individuals, both of which were assessed by the FOT, plethysmography and spirometry. An equivalent electrical circuit model was also used to quantify biomechanical changes and to gain physiological insight.

**Results and discussion:**

The CF adults presented an increased total respiratory resistance (p < 0.0001), increased resistance curve slope (p < 0.0006) and reduced dynamic compliance (p < 0.0001). In close agreement with the physiology of CF, the model analysis showed increased peripheral resistance (p < 0.0005) and reduced compliance (p < 0.0004) and inertance (p < 0.005). Significant reasonable to good correlations were observed between the resistive parameters and spirometric and plethysmographic indexes. Similar associations were observed for the reactive parameters. Peripheral resistance, obtained by the model analysis, presented reasonable (R = 0.35) to good (R = 0.64) relationships with plethysmographic parameters.

**Conclusions:**

The FOT adequately assessed the biomechanical changes associated with CF. The model used provides sensitive indicators of lung function and has the capacity to differentiate between obstructed and non-obstructed airway conditions. The FOT shows great potential for the clinical assessment of respiratory mechanics in adults with CF.

## Introduction

Pulmonary disease, with a mortality rate of 85%, is the major cause of morbidity and mortality in individuals with Cystic Fibrosis (CF) [1]. CF care has dramatically improved over the last decades. In 2011, more than 48% of CF patients in the Patient Registry of the CF Foundation were adults, and this number continues to grow [1]. The emergence of a larger population of adults living with CF necessitates further understanding of the overall changes in their lung function. Such studies will provide essential information for clinical care and research. Understanding the mechanisms of lung injury may guide choices in clinical care and the development of new therapies.

The forced expiratory volume in 1 second (FEV_1_) has been used as a predictor of survival and has been widely adopted in clinical studies of lung function in children, adolescents and adults with CF [1]. However, changes in respiratory mechanics are not always detected by this test [2]. People of all ages with CF have some lung damage, even when their lung function is normal as evaluated by the FEV_1_, and this measurement has the limitation of being insensitive to early changes in lung disease [1].

The Forced Oscillation Technique (FOT) offers a simple and detailed approach to investigate the mechanical properties of the respiratory system and represents the current state-of-the-art assessment of lung function [3,4]. This method consists of the application of sinusoidal signals during normal respiration by means of an external pressure generator, allowing for the measurement of respiratory system impedance. The main advantage of the FOT is the simplicity of the test administration, which requires little cooperation from the patient and involves the production of parameters describing the resistive and reactive properties of the respiratory system that are complementary to traditional pulmonary assessment methods [3-5]. These characteristic were important to explore lung function in children with CF [6-11]. Recently, this technique was successfully applied in our laboratory to study the early respiratory changes in smokers [12] and patients with sarcoidosis [13] and systemic sclerosis [14]. Compartmental models of the multi-frequency impedance allowed a detailed description of the human respiratory system by equivalent electrical circuits [15,16]. These models allow us to gain additional insight into the anatomical or pathophysiological changes that occur in respiratory diseases. In addition to being useful in furthering our understanding of respiratory biomechanical function, these model parameters could improve the detection, diagnosis, and treatment of different respiratory diseases.

Therefore, the FOT has the potential to increase our knowledge about the biomechanical abnormalities of adults with CF. However, as noted in a recent review, there are no reports in the literature evaluating the FOT in adults with CF, and further research is needed in this area before the FOT can be widely used in clinical practice [17].

In this context, the purpose of the present study was to analyze the changes in the resistive and reactive biomechanical properties of the respiratory system in adults with CF.

## Materials and methods

### Patients and study design

This study utilized a cross-sectional design involving two groups of subjects: a group of CF adults aged 19–43 years and a group of controls. The diagnosis of CF was based on the presence of at least two of the following criteria: sweat chloride concentration >60 mEq/mL; two clinical features consistent with CF [18]; or genetic testing demonstrating two mutations associated with CF [19].

The CF group was composed of patients who had no reports of smoking, hemoptysis and/or recent hemopysis, thoracic surgery, cold or flu in the last month, or recent worsening respiratory infection (within 2 weeks of the exams). The control group was composed of individuals who had no reports of flu or cold during the last month, respiratory symptoms, lung disease, cognitive impairment, heart disease, history of smoking, hospitalization in the past six months or spirometric abnormalities. Demographic data, including age, height and weight, were obtained from each subject at the time of the procedures.

This study was approved by the Medical Research Ethics Committee of the State University of Rio de Janeiro. The work was performed in accordance with The Declaration of Helsinki. Informed consent was obtained from all volunteers before inclusion in the study.

### Spirometry

A closed circuit spirometer (Warren E. Collins, Inc., Braintree, MA, USA) was used to measure the forced vital capacity (FVC), FEV_1_, FEV_1_/FVC ratio, expiratory flow between 25% and 75% of the FVC (FEF_25%-75%_) and FEF_25%-75%_/FVC ratio. These parameters were expressed as absolute values and as a percentage of the predicted values (% of predicted), and the reference values were obtained from the equations of Knudson et al. [20]. These measurements were conducted according to the recommendations of the American Thoracic Society/European Respiratory Society [21]. An obstructive ventilatory defect was defined as an FEV_1_/FVC ratio below the normal lower limit, as recommended in previous guidelines [21,22].

### Plethysmography

Using a constant volume and variable pressure plethysmograph (Warren E. Collins, Inc., Braintree, MA, USA), we evaluated the total lung capacity (TLC), functional residual capacity (FRC) and residual volume (RV), as well as their relationships (RV/TLC and FRC/TLC). The airway resistance (Raw) and specific airway conductance (SGaw) were also measured [23]. The reference values were obtained from Goldman and Becklake [24].

### Forced Oscillation

The respiratory system impedance was measured between 2 and 32 Hz with 2-Hz increments using a pseudorandom noise forced oscillation system that was built in our laboratory [12,14,25]. Pressure oscillations, which were produced by a loudspeaker coupled to the respiratory system by means of a mouthpiece, were applied with amplitude of approximately 1 cmH_2_O. The resulting flow (V’) and pressure (P) signals were measured near the mouth by means of a pneumotachograph and a pressure transducer, respectively. The common mode rejection ratio of the airflow measurement system (65.2 dB) was adequate for these studies [26]. The frequency response of the pressure measurement system is flat in the used frequency range. The small errors associated to the attenuation and phase shift introduced by the flow measurement system was corrected digitally using calibration curves obtained by means of the analysis of a reference mechanical load [27].

The respiratory system impedance (Zrs(f)) was estimated from the Fourier analysis of these signals, as described by equation (1).

1 $$ {\mathrm{Z}}_{\mathrm{rs}}\left(\mathrm{f}\right) = \frac{\overline{{\mathrm{G}}_{\mathrm{PV}}}}{\overline{{\mathrm{G}}_{\mathrm{VV}}}} $$

Where $\overline{{\mathrm{G}}_{\mathrm{PV}}}$ and $\overline{{\mathrm{G}}_{\mathrm{VV}}}$ are the mean crosspectrum between pressure and flow and mean autospectrum of flow, respectively. Three FOT exams were performed; each exam lasted approximately 16 seconds, and the exams were separated by one-minute intervals. During the exams, each individual wore a nose clip to prevent air escape and breathed calmly through a silicone mouthpiece. The subjects remained seated with their chests straight and their heads in a neutral position relative to the device. Additionally, the individuals firmly supported their extrathoracic airways to minimize the potential confounding effects of airway wall flow shunting [3-5]. To express the amount of noise on the pressure and flow signal, a coherence function was calculated at each investigated frequency. Only exams with a coherence function between 0.9 and 1.0 were retained [14,28]. The final result of the tests was estimated by calculating the average of three satisfactory exams.

Linear regression analysis of the resistance values in the frequency range between 4 and 16 Hz was used to determine the intercept resistance (R0) and the slope of the resistive component of the impedance (S). R0 is associated with the airway and tissue Newtonian resistance plus the delayed airway resistance resulting from gas redistribution [14]. S reflects both spatial and temporal ventilatory non-homogeneity [28,29]. The value of Rrs measured in 4 Hz (R4) was also analyzed.

The reactance in the frequency range between 4 and 32 Hz was used to measure the resonant frequency (fr), the respiratory system mean reactance (Xm) [30], and the area under the negative part of the reactance curve (Ax) [15]. Fr is defined as the lowest frequency at which the reactance crossed 0 from negative to positive. Fr reflects the airway heterogeneity changes and tissue changes. Xm is usually related to respiratory system non-homogeneity [31]. Ax is related to respiratory compliance, and reflects changes in the degree of peripheral airway obstruction [15]. The dynamic compliance of the respiratory system (Cdyn) was estimated using the respiratory reactance at 4 Hz (X4Hz) and the equation Cdyn = −1/(2πfX4Hz) [32]. The same frequency was used to evaluate the absolute value of the respiratory impedance (Zrs). This parameter is associated with the work performed by the respiratory muscles to overcome resistive and elastic loads, promoting the movement of air in the respiratory system [14,33].

### Respiratory impedance model

**Figure 1:**
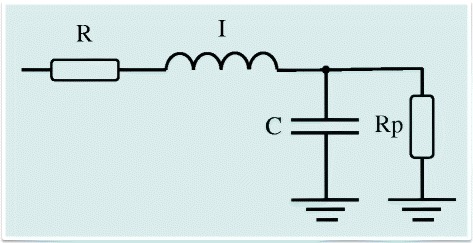
**Electrical representation of a two-compartment model used to analyze respiratory impedance.** Resistance, inductance and capacitance are the analogs of mechanical resistance, inertance and compliance, respectively. R is analogous to central airway resistance and Rp describes peripheral resistance, I is associated with airway, gas and tissue inertance, while C is related with alveolar compliance. This analysis also evaluated the total resistance (Rt = R + Rp), which included the effects of central and peripheral airways.

Compartmental model analysis was performed using the extended RIC (eRIC) model (Figure 1), in which R is analogous to central airway resistance, Rp describes peripheral resistance, I is associated with lung inertance and C is associated with alveolar compliance [16]. This model is proposed as an improvement to the basic RIC model [4,15]. Specifically, the added peripheral resistance Rp allows for observation of the frequency dependence of typical real impedance data, which is beyond the capability of the RIC model. This additional component describes the resistance presented by the small airways of the respiratory system. We also evaluated the total resistance (Rt = R + Rp), which includes the effects of the central and peripheral airways. Model parameters were estimated using the Levenberg-Marquardt algorithm to determine the set of coefficients of the nonlinear model that best represents the input data set in the least squares sense. Along with the corresponding model estimates, this analysis also provided the evaluation of the total error value, which is an overall measure of “goodness of fit” for the model. Herein, this parameter is defined as the square root of the sum of the real and imaginary squared impedance estimation errors. The mean relative distance from the model and measured resistance and reactance values was also measured [34,35].

### Statistical analysis

Statistical analysis was performed using STATISTICA 5.0 (StatSoft, Inc., Tulsa, OK, USA) and Microcal Origin version 6.0 (Microcal Software, Inc. Ostend, Belgium). First, the Shapiro-Wilks W test was used to analyze whether the data were normally distributed. Student’s t test was used to analyze normally distributed data, and the Mann–Whitney U test and Wilcoxon Matched Pairs Test were used when the data were not normally distributed. The results with p values < 0.05 were considered statistically significant.

To measure the overall agreement between the variables related to the FOT, respiratory modeling, spirometry, plethysmography and diffusion, we calculated Pearson’s correlation coefficient for the whole group of studied volunteers. These correlations were classified as follows [36]:

- Small or no correlation: between 0 and 0.25 (or −0.25);
- Reasonable correlation: between 0.25 and 0.50 (or −0.25 to −0.50);
- Moderate to good correlation: between 0.50 and 0.75 (or −0.50 to −0.75);
- Very good to excellent correlation: greater than 0.75 (or −0.75).

## Results

A total of 23 control subjects and 27 CF patients were included in the study. The patient characteristics are shown in Table 1. The biometric characteristics of the two studied groups were well matched, and there were no significant differences between the groups.

**Table 1:** **Anthropometric characteristics of the studied subjects (mean ± standard deviation)**

	**Control group (n = 23)**	**Cystic fibrosis (n = 27)**	**p**
Gender (F/M)	14/09	16/11	-
Age (years)	25.6 ± 3.1	25.0 ± 5.7	ns
Weight (kg)	64.4 ± 12.8	59.4 ± 13.7	ns
Height (cm)	167.1 ± 8.9	166.8 ± 8.9	ns
BMI (kg/m^2^)	23.0 ± 2.8	21.2 ± 3.8	ns

The average time of CF diagnosis was 12.4 years. Mutations in the cystic fibrosis transmembrane conductance regulator (CFTR) gene were distributed as follows: 56 (52.8%) patients had two severe mutations (classes I–III and VI); 28 (26.4%) patients had one severe mutation and one unknown mutation; 11 (10.4%) patients had one severe and one mild mutation (class IV–V); and 11 (10.4%) patients had no known mutations on both alleles. In 5 (4.7%) patients, no mutations were identified, and in 6 (5.7%) patients, mutation-analysis was not available at the time of study inclusion; however, all 11 of these patients had previous repeat pilocarpine-iontophoresis sweat tests with pathological results to establish their CF diagnoses. In this sample, 13 (48.1%) patients were chronically infected by *Pseudomonas aeruginosa*; 2 (7.4%) patients were chronically infected by the *Burkholderia cepacia* complex; 4 (14.8%) patients were chronically infected by both *P. aeruginosa and B. cepacia* complexes; and 6 (22.2%) patients had no chronic pulmonary infection. The prevalence of pancreatic insufficiency and diabetes mellitus was 63% and 11.1%, respectively.

A significant reduction was observed in all of the studied spirometric parameters in the CF group (Table 2). An obstructive ventilatory defect was diagnosed in 23 cases (85.2%). Regarding the plethysmographic parameters, no patient had a TLC below the normal lower limit. There were no significant differences in the TLC and FRC, whereas the RV significantly increased. The Raw was significantly increased in CF patients (p < 0.0001), whereas the SGaw was significantly reduced (p < 0.0001).

**Table 2:** **Spirometric, plethysmografic and diffusion characteristics of the studied subjects**

	**Control group (n = 23)**	**Cystic fibrosis (n = 27)**	**p**
Spirometry			
FVC (L)	4.2 ± 1.0	3.5 ± 1.3	0.03
FVC (%)	101.7 ± 13.0	84.1 ± 2 5.4	0.004
FEV_1_ (L)	3.7 ± 0.8	2.3 ± 1.1	0.0001
FEV_1_ (%)	105.2 ± 13.8	65.8 ± 28.6	0.0001
FEV_1_/FVC	88.7 ± 4.4	64.4 ± 13.9	0.0001
FEF_25–75%_ (L)	4.6 ± 1.3	1.6 ± 1.3	0.0001
FEF_25–75%_ (%)	113.3 ± 26.7	39.1 ± 30.0	0.0001
FEF_25–75%_/FVC	110.7 ± 23.8	41.8 ± 26.6	0.0001
Plethysmography			
TLC (L)	5.6 ± 1.0	5.7 ± 1.4	ns
TLC (%)	99.4 ± 12.2	100.2 ± 19.2	ns
FRC (L)	2.7 ± 0.6	3.4 ± 1.3	ns
FRC (%)	88.3 ± 17.4	100.6 ± 31.2	ns
RV (L)	1.6 ± 0.5	2.3 ± 1.4	0.008
RV (%)	101.5 ± 33.0	149.0 ± 95.2	0.007
RV/TLC	27.8 ± 8.7	39.2 ± 15.9	0.004
RV/TLC (%)	100.8 ± 28.6	144.4 ± 59.9	0.001
FRC/TLC	49.9 ± 8.0	56.3 ± 11.1	ns
Raw (cmH_2_O/L/s)	0.73 ± 0.28	1.94 ± 1.31	0.0001
SGaw (cmH_2_O^−1^ s^−1^)	0.49 ± 0.24	0.19 ± 0.12	0.0001

**Figure 2:**
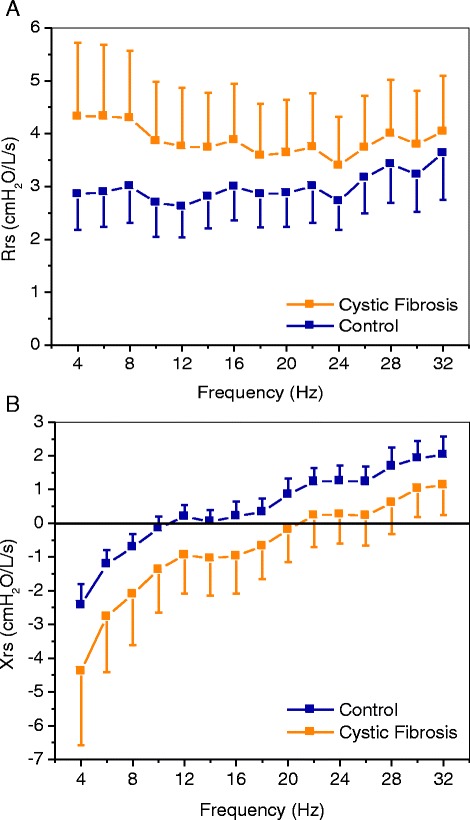
**Mean course of respiratory resistance (A) and reactance (B) as a function of frequency in the normal subjects and in adult patients with cystic fibrosis.**

Figure 2 shows the mean curves of the respiratory resistance (Rrs) and reactance (Xrs) as functions of the frequencies in normal and CF subjects. The Rrs curve in CF adults was significantly different from the control curve (Figure 2A; p < 0.0001), whereas the Xrs curves displayed a smaller difference between the groups (Figure 2B; p < 0.03).

**Figure 3:**
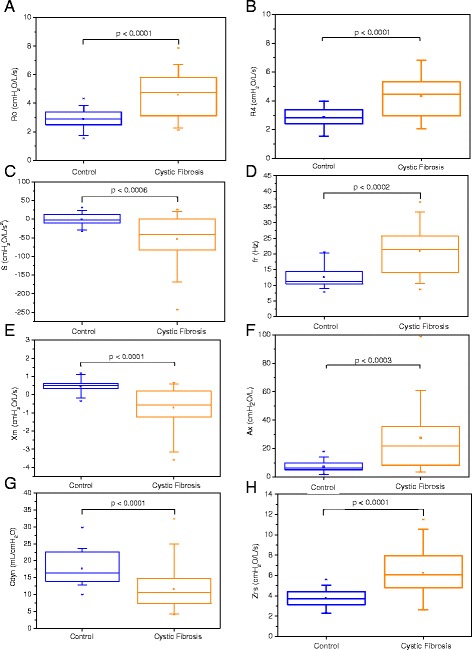
**Comparative analysis of the resistive parameters obtained from groups of adults with Cystic Fibrosis and control: Respiratory system resistance (R0; Figure A), resistance in 4 Hz (R4; Figure B), slope of respiratory resistance (S; Figure C).** Similar analysis for reactive parameters are also described: resonant frequency (fr; Figure **D**), mean reactance (Xm; Figure **E**), reactance area (Ax; Figure **F**), dynamic compliance (Cdyn; Figure **G**) and the impedance modulus (Zrs; Figure **H**). The top and the bottom of the box plot represent the 25th- to 75th-percentile values, while the circle represents the mean value, and the bar across the box represents the 50th-percentile value. The whiskers outside the box represent the 10th-to 90th-percentile values.

Adults with CF presented an increased R0 (Figure 3A; p < 0.0001) and R4 (p < 0.0001), whereas S was significantly more negative (p < 0.0006). The presence of CF was associated with higher fr values (p < 0.0002), more negative Xm values (p < 0.0001), more positive Ax (p < 0.0003) and lower Cdyn values (p < 0.0001). In contrast, the Zrs values were higher in CF patients (p < 0.0001).

**Figure 4:**
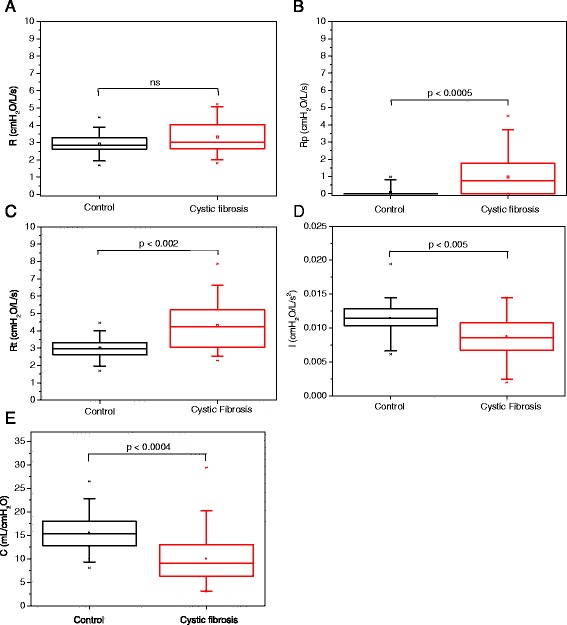
**Influence of cystic fibrosis in adult patients on parameter values estimated from the model described in Figure** **1** **: central airway resistance (R; Figure A), peripheral resistance (Rp; Figure B), total resistance (Rt; Figure C), lung inertance (I; Figure D) and alveolar compliance (C; Figure E).** The top and the bottom of the box plot represent the 25th- to 75th-percentile values, while the circle represents the mean value, and the bar across the box represents the 50th-percentile value. The whiskers outside the box represent the 10th-to 90th-percentile values.

The changes in the parameters of the eRIC model associated with CF are described in Figure 4. The mean total error in the model estimates was 0.13 cmH_2_O/L/s and 0.10 cmH_2_O/L/s in the control group and FC group, respectively. The relative distance from the model and measured resistance and reactance values was 3.5 ± 1.1% in controls and 2.1 ± 0.7% in patients. There was no significant change in the R values, whereas the Rp significantly increased in CF (Figures 4A and B, respectively). A significant increase in the Rt occurred in CF (Figure 4C). Significant reductions in I and C values were observed (Figures 4D and 4E, respectively).

In general, moderate to good relationships between the resistive FOT parameters (R0, R4 and S) and spirometric indexes (Table 3) were observed. The reactive parameters (fr, Ax and Xm), presented better relationships; specifically, the Cdyn presented excellent correlations with the FVC (L) and FEV_1_ (L) (Table 3). Considering the associations obtained from compartmental model analysis, it was observed that R was not related with the spirometric parameters, whereas Rp and Rt presented moderate to good relationships. On the other hand, C showed a very good direct correlation with the FVC (L) and FEV_1_ (L).

**Table 3:** **Correlation coefficient and significance level of the analysis including FOT, compartmental model and spirometric exams**

	**FVC (L)**	**FVC (%)**	**FEV**_**1**_ **(L)**	**FEV**_**1**_ **(%)**	**FEV**_**1**_ **/FVC**	**FEF**_**25–75%**_ **(L/s)**	**FEF**_**25–75%**_ **(%)**	**FEF/FVC**
FOT parameters								
R0 (cmH_2_O/L/s)	−0.48**	ns	−0.58*	−0.44*	−0.53**	−0.55**	−0.50**	−0.48**
R4 (cmH_2_O/L/s)	−0.51***	−0.41**	−0.66****	−0.61****	−0.64****	−0.68****	−0.66****	−0.61****
S (cmH_2_O/L/s^2^)	0.61***	0.56**	0.74****	0.74****	0.79****	0.72****	0.73****	0.70****
fr (Hz)	−0.63***	−0.48*	−0.73****	−0.64**	−0.65**	−0.69***	−0.67**	−0.61**
Xm (cmH_2_O/L/s)	0.69****	0.63****	0.78****	0.74****	0.77****	0.69****	0.69****	0.68****
Ax (cmH_2_O/L)	−0.59****	−0.59****	−0.72****	−0.74****	−0.76****	−0.65****	−0.66****	−0.63****
Cdyn (L/cmH_2_O)	0.80****	0.65***	0.81****	0.71***	0.59*	0.70****	0.64***	0.56**
Zrs (cmH_2_O/L/s)	−0.69****	−0.53**	−0.76****	−0.66***	−0.70****	−0.67**	−0.63***	−0.60**
Compartmental model								
R (cmH_2_O/L/s)	ns	ns	ns	ns	ns	ns	ns	ns
Rp (cmH_2_O/L/s)	−0.50***	−0.55****	−0.64****	−0.70****	−0.74****	−0.59****	−0.60****	−0.59****
Rt (cmH_2_O/L/s)	−0.43**	−0.35*	−0.59****	−0.56****	−0.63****	−0.62****	−0.61****	−0.58****
I (cmH_2_O/L/s^2^)	ns	ns	0.32*	ns	0.51****	0.36**	0.40**	0.44**
C (mL/cmH_2_O)	0.76****	0.68****	0.79****	0.74****	0.60****	0.67****	0.63****	0.52****

FOT: Forced Oscillation Technique; ns: non-significant; *p < 0.05; **p < 0.01; ***p < 0.001 and ****p < 0.0001.

Significant reasonable to good correlations were observed between the resistive parameters (R0, R4 and S) and plethysmographic indexes (Table 4). Similar associations were observed for the reactive parameters (fr, Xm, Ax, Cdyn and Zrs). The strongest relationships were observed for Xm and Zrs with RV/TLC (R = −0.69; p < 0.0001). Via the model analysis, it was observed that R was not correlated with the plethysmographic parameters, whereas Rp presented reasonable (R = 0.35) to good (R = 0.64) relationships with the parameters. C showed a good inverse correlation with the RV/TLC (R = −0.65).

**Table 4:** **Correlation coefficient and significance level of the analysis including FOT, compartmental model and plethysmographic exams**

	**TLC (L)**	**TLC (%)**	**FRC (L)**	**FRC (%)**	**RV (L)**	**RV (%)**	**RV/TLC**	**RV/TLC (%)**	**FRC/TLC**	**Raw (cmH**_**2**_ **O/L/s)**	**SGaw (cmH**_**2**_ **O** ^**−1**^ **s** ^**−1**^ **)**
FOT Parameters											
R0 (cmH_2_O/L/s)	ns	ns	ns	ns	0.33*	0.36**	0.50***	0.48***	0.30*	0.56****	−0.52***
R4 (cmH_2_O/L/s)	ns	ns	ns	ns	0.29*	0.33*	0.46***	0.44**	ns	0.54****	−0.51***
S (cmH_2_O/L/s^2^)	ns	ns	−0.33*	−0.46***	−0.55****	−0.58****	−0.66****	−0.67****	−0.50***	−0.66****	0.52***
fr (Hz)	ns	ns	ns	ns	0.36*	0.37**	0.58****	0.56****	0.41**	0.61****	−0.56****
Xm (cmH_2_O/L/s)	ns	ns	−0.36*	−0.45**	−0.57****	−0.59****	−0.69****	−0.69****	−0.54****	−0.65****	0.54****
Ax (cmH_2_O/L)	ns	ns	0.32*	0.45**	0.54****	0.56****	0.67****	0.66****	0.53****	0.64****	−0.49***
Cdyn (L/cmH_2_O)	0.34*	ns	ns	ns	−0.38**	−0.42**	−0.61****	−0.59****	−0.33*	−0.57****	0.38**
Zrs (cmH_2_O/L/s)	ns	ns	0.29*	0.42**	0.53****	0.57****	0.69****	0.67****	0.48***	0.64****	−0.53****
Compartmental model											
R (cmH_2_O/L/s)	ns	ns	ns	ns	ns	ns	ns	ns	ns	ns	ns
Rp (cmH_2_O/L/s)	ns	ns	0.35*	0.46***	0.52***	0.54****	0.60****	0.61****	0.52***	0.64****	−0.49***
Rt (cmH_2_O/L/s)	ns	ns	ns	ns	0.30*	0.33*	0.42**	0.41**	ns	0.47***	−0.44**
I (cmH_2_O/L/s^2^)	ns	ns	−0.28*	ns	ns	ns	−0.29*	−0.30*	ns	−0.38**	0.38**
C (mL/cmH_2_O)	0.31**	ns	ns	ns	−0.42**	−0.45**	−0.65****	−0.64****	−0.41**	−0.58****	0.43**

FOT: Forced Oscillation Technique; ns: Non-significant; *p < 0.05; **p < 0.01; ***p < 0.001 and ****p < 0.0001.

## Discussion

This study is the first to systematically evaluate the use of the forced oscillation technique in adults with CF. Two major findings were observed: 1) adult patients with CF presented increased Rrs and more negative Xrs values, and 2) the FOT parameter alterations were correlated with the standard pulmonary function analysis methods.

In agreement with other studies, we observed that the majority of adults with CF had obstructive airway disease [17,37]. In CF, several mechanisms contribute to airflow limitation, including chronic inflammation, increased smooth muscle in the airway walls, mucus hypersecretion with the formation of viscous secretions, and bronchiectasis [38,39]. The average FEV_1_ in our patients was 65.8%, similar to that observed by Yankaskas et al. [40] in an American, adult CF population. Compared with the controls, the CF patients in the present study demonstrated a significant increase in both the RV and the RV/TLC ratio. Interestingly, the clinical status of adults with CF is negatively correlated with the amount of air trapping in poorly ventilated areas of the lungs [37]. Thus, patients with worse clinical conditions tend to have greater air trapping.

In contrast to the relatively uniform resistance values in the different frequencies observed in healthy subjects, the resistance was increased at lower frequencies and decreased with increasing frequency applied via the FOT in CF patients (Figure 2). Thus, the negative dependence of the resistance on the frequency in patients with airway obstruction may have resulted from a redistribution of alveolar gas due to ventilation heterogeneity or changes in airway compliance [28]. In the present study, the abnormalities in the resistance curve may be related to airway obstruction, which was detected in 85.2% of adults with CF.

Resistances measured between 4–32 Hz are related to the airway and tissue Newtonian resistance plus the delayed airway resistance resulting from gas redistribution. R0 is an extrapolation to the intercept, estimating how the cited properties work at low frequencies. Thus, this parameter, as well as R4, does not include tissue viscoelastic properties, but it is important to evaluate how low-frequency Newtonian and delayed airway resistance changes in disease. R0 is related to the total resistance of the respiratory system, and can be proposed as an index to evaluate the degree of obstruction and to assess the reversibility of the airways [28]. Because there are no other studies of the FOT in adults with CF, it is reasonable to compare our results with those of other studies conducted in patients with airway obstruction due to other causes. The average R0 value (Figure 3A; 4.71 ± 1.52 cmH_2_O/L/s) was similar to those obtained in adults with asthma and patients with COPD with moderate airway obstruction [28,41,42]. R0 and R4 also include the effect of the central airway resistance. Thus, the significant increase of these parameters may also include the involvement of large airways in adults with CF. Despite the initial damage that occurs in the small airways, the central and hilar bronchi are always involved when bronchiectasis is present [43]. The alterations of R0 and R4 were inversely correlated with forced expiratory flows (Table 3) and directly related with pulmonary volumes and airway obstruction (Table 4). This allowed us to infer that these parameters represent alterations of the central and peripheral airways and are also sensitive to air trapping (associated with the RV and the RV/TLC ratio), which is a typical abnormality found in CF. It is interesting to note that, while the associations of R4 were higher than that observed in R0 with spirometric parameters (Table 3), the associations of R0 were higher than that observed for R4 with plethysmographic values (Table 4).

In adults with CF, the average S value was significantly more negative than in healthy subjects (Figure 3C). This finding is in agreement with a previous study of asthma that also evaluated patients with airflow limitation [41]. S is associated with the non-homogeneity of the respiratory system [28]. Among the causes that can lead to non-homogeneity in the respiratory system in CF, the most important cause is the uneven distribution of pulmonary lesions. In CF patients, there is a preference for regional involvement, which is most pronounced in the upper lung zones [43]. In agreement with this interpretation, Table 3 shows that S was associated with the spirometric parameters, mainly those sensitive to both airway closure and airway narrowing (FEV1), and that representing alterations of the central (FEV1/FVC) and the small (FEF25–75%) airways. Additional support for this interpretation is shown in Table 4, in which S is shown to be correlated with residual volume abnormalities and airway obstruction.

Figure 2B shows that CF in adult patients introduced more negative Xrs values. According to Clement et al. [44], the Xrs may contribute to the discrimination between healthy individuals and patients with respiratory symptoms. As a result of the Xrs changes, adults with CF had higher fr values (Figure 3D) and more negative Xm values (Figure 3E). Similarly, Hellinckx et al. [45] also observed more negative reactance values in children with CF. Whereas S is associated with non-homogeneity in terms of resistance distribution, Xm describes non-homogeneity in terms of the reactive properties of the respiratory system. Ax (Figure 3F) presented smaller discrimination than that presented by Xm and Cdyn (p < 0.0001). This parameter also presented smaller association with spirometric (Table 3) and plethysmographic (Table 4) parameters than Xm. The fr value may be a sensitive marker of incipient airway obstruction [25]. In line with this hypothesis, an inverse association was observed with the spirometric parameters (Table 3), as well as direct relationships with the residual volume and airway obstruction (Table 4). Xm was also associated with the spirometric parameters (Table 3), the residual volume and airway obstruction (Table 4). The association between Xm and RV/TLC is interesting, since this ratio is supposed to be of prognostic value. Higher fr and more negative Xm values in patients with airway obstruction may also be related to reduced lung compliance, which results in a predominance of the negative phase of the reactance curve, as can be observed in Figure 2B.

The viscoelastic behavior of lung tissue dominate over the low-frequency range (below about 2 Hz) [4]. For frequencies below 5 Hz, the effect of the inductance is negligible [46,47]. In the medium-frequency range (2–40 Hz), reactance transitions at the resonance frequency from dominance by the tissue elastic properties at low frequencies to dominance by the inertial properties of the gas in the airways at higher frequencies [4]. In this study, the Cdyn was measured at a frequency of 4 Hz, at which the impedance mainly reflects the elastic properties. Hyatt et al. [23] suggested that the reduced Cdyn values may be associated with decreased lung compliance or increased airway resistance. Considering that dynamic lung compliance is a major component of the Cdyn, the most likely causes for their reduction in CF patients (Figure 3G) include a reduced number of functioning small airways, increased airflow resistance, airway inflammation, increased secretions and parenchymal inflammation/fibrosis [17]. This interpretation is supported by the associations observed with the spirometric (Table 3) and plethysmographic (Table 4) indexes of airway obstruction.

The respiratory impedance module is related to the total mechanical load of the respiratory system and describes the effects of the respiratory system resistance and compliance. This parameter is related to the work performed by the respiratory muscles to overcome resistive and elastic loads to promote the movement of air in the respiratory system [33]. It is also related to fatigue and breathlessness, which are among the most important symptoms in predicting the quality of life in respiratory patients. The increase in Zrs values observed in CF (Figure 3H) may be due to several factors, including the increase in respiratory resistance, associated with the reduction of airway caliber, and the reduced dynamic compliance. The associations observed with airway obstruction (Tables 3 and 4) and residual volume (Table 4) are consistent with this interpretation. Similar to Xm, the correlation observed between Zrs and RV/TLC is clinically interesting, since this ratio is supposed to be of prognostic value.

The compartmental model analysis may contribute to a better understanding of the biomechanical abnormalities of CF in adults. Figure 4 shows that CF does not introduce significant increases in the central resistance values (Figure 4A), whereas both the peripheral (Figure 4B) and total resistance (Figure 4C) values significantly increased.

R was not associated with spirometric or plethysmographic parameters (Tables 3 and 4), whereas Rp presented a significant inverse association with the forced expiratory flow (Table 3), and direct relationships with the Raw and the RV (Table 4). Rt presented similar associations, with slightly reduced values. These results are in line with the pathophysiology of CF [17], providing additional evidence that the harmful effects of CF-related dysfunctions are particularly manifested in the distal airways [17,19,45]. These small airways, by nature of their size and architecture, are particularly susceptible to the effects of airway wall inflammation and obstruction [17,19].

Respiratory inertance mainly reflects the mass of gas that is moved during spontaneous ventilation. Since this parameter is proportional to the ratio of airway length to diameter [48], it might be expected that the CF patients would have a higher inertance. However, the estimates of respiratory inertance using the eRIC model showed reduced values in CF patients (Figure 4D). These results are consistent with Diong et al. [16], which observed a decrease in inertance in patients diagnosed with mild obstructive lung disease. Similar results were also obtained by Michaelson et al. [49] and Hayes et al. [50] in obstructive patients, by Ionescu and de Keyser [51] using several different models to evaluate COPD patients and by Iwatsubo et al. [52] in subjects displaying moderate airway obstruction. It is important to point out that Iwatsubo et al. [52] used the standard and head generator methods, which indicates that the reduction of inertance is not associated with upper airway shunt. The reasons for this behavior may be interpreted using the concepts of choke points [53] and apparent compliance [54]. Under normal conditions, inertance reflects the inertial properties of the entire respiratory system. However, when obstruction is present, the oscillatory signal cannot pass through these choke points and reach the alveoli, which prevent the FOT from being able to probe the lung beyond the choke point where obstruction occurs. This way, FOT measurements reflects the mechanical properties of airways proximal to the choke points. This result in a reduction of the apparent mass of the gas accelerated by the FOT signal, and consequently a reduction in the pressure required for gas acceleration, and in the measured inertance. Thus, we may speculate that the airway obstruction may result in an apparent inertance, similar to the process observed in the apparent compliance. Theoretically, these changes would be associated with retained air within the obstructed lung units, which would increase RV and FRC. This hypothesis was confirmed by the inverse association observed between inertance and the FRC and RV/TLC (Table 4). It is also consistent with the direct association observed between inertance and spirometric indices of obstruction (Table 3).

As can be observed in Figure 4E, the analysis based on the eRIC model also revealed reduced values of compliance. These results clearly reflect the CF physiopathology, which includes airway inflammation, increased secretions and parenchymal inflammation/fibrosis [17]. This accurate description of the CF physiopathology afforded by the Cdyn and C allows us to infer that the FOT might be an alternative method to quantitatively and noninvasively assess the elastic properties of the respiratory system in these individuals, thus avoiding the use of invasive techniques such as those using an esophageal balloon.

One may argue about the redundancy of the evaluation of Cdyn and C. Note that these parameters are estimated by two very different methods. The comparison of the parameters obtained by these two different approaches help to understand what is the better way to evaluate the elastic properties of the respiratory system. The performance of these parameters, described in Tables 3 and 4, indicates that they present very similar results. Considering that the estimative of Cdyn is simpler, do not demanding the computational effort to fit a model, it seems more adequate than C.

Similarly to other functional assessment techniques, the limitations and consequences of the FOT must be recognized. The FOT is subject to the influence of upper airway shunting; this must be carefully controlled for when using this method. The resulting effect is a reduced impedance measurement relative to its actual value. This effect becomes progressively stronger as the respiratory resistance increases, as is the case for highly obstructive patients. Because we are studying patients with relatively low resistance values (Figures 2, 3 and 4) compared with typical obstructive patients [41,42], this limitation did not present a notable problem in this study. In addition, the shunt effect was minimized by asking the patients to firmly support their cheeks and mouth floors [3,4]. A second limitation arises from the process of spontaneous breathing, which introduces both random and systematic errors [3,4]. These errors were minimized in the present study using excitation frequencies (4–32 Hz) at least 20 times higher than those present in the spontaneous ventilation process (approximately 0.2 Hz). These errors can be easily evaluated using the coherence function (γ^2^) between the pressure and airflow signals [3,4]. In this study, any time the coherence value computed for any of the studied frequencies was smaller than a minimal threshold (0.90), the maneuver was not considered valid, and the examination was repeated [33,41,42].

The multiple breath inert gas washout (MBW) test assesses efficiency of gas mixing processes by following the expiration of inert tracer gas from the lungs during tidal breathing [17]. Since there is evidence that this method may help to assess the uniformity of ventilation distribution and to detect early lung disease, there has been considerable investigation as to the role of MBW in CF management [17,55-57]. The use of MBW as a gold standard may contribute to provide further information concerning the use of FOT in adults with CF. This hypothesis warrants further study.

Fitting mathematical models may help to obtain parameters describing lung structure and function. However, there are numerous models, and different models may be fitted by the same impedance data [4]. It is important to point out that more complicated models would not allow statistically reliable parameter estimates [58,59]. Thus it is not possible to include every known component of a complicated system in a mathematical model. Therefore, the choice of model structure requires a decision about which components of the real system are important for the purpose at hand, and which components can be safely ignored [60]. To minimize the problem of model complexity, a simple four-element compartmental model was used for interpreting forced oscillation measurements [16]. However, we believe that studies focusing on fractional order impedance models [46] and more complex models associated with a wider frequency range [4,46,48] could contribute with new information concerning the effects of CF in adults and should therefore be addressed in future research.

Partial correlation coefficients and multiple step wise regression analysis may help to further clarify the physiological meaning and the association between each of the studied parameters and the physiological changes. We believe that these analyses in a higher number of subjects deserve further studies.

A critical analysis of the limitations of the present study is also necessary. First, the present report was a cross-sectional study of clinically stable patients. Future studies should incorporate the analysis of the FOT relative to the long-term results and their role in exacerbations. Second, the volunteers were recruited at one center, which might hinder the generalizability of the results.

In conclusion, in this study, we present evidence of increased total respiratory resistance and reduced ventilation homogeneity and dynamic compliance in adults with CF. The eRIC model analysis showed abnormally increased peripheral resistance values. This analysis contributed to the elucidation of the biomechanical changes in these patients. The FOT associated with the eRIC model is a non-invasive, simple and radiation-free method for the detection of biomechanical abnormalities. Thus, this method has promising applications as a complementary pulmonary function test in adult CF patients.
